# Antibody treatment of hepatocellular carcinoma: a review of current and emerging approaches

**DOI:** 10.3389/fimmu.2025.1533874

**Published:** 2025-06-20

**Authors:** Sherif A. El-Kafrawy, Mostafa S. Elkafrawy, Esam I. Azhar, Anwaar Saeed, Ashraf A. Tabll

**Affiliations:** ^1^ Special Infectious Agents Unit- BioSafety Level 3 (BSL3), King Fahd Medical Research Center, King Abdulaziz University, Jeddah, Saudi Arabia; ^2^ Department of Medical Laboratory Sciences, Faculty of Applied Medical Sciences, King Abdulaziz University, Jeddah, Saudi Arabia; ^3^ Faculty of Medicine, Menoufia University, Shebin El-Koam, Egypt; ^4^ Department of Medicine, Division of Hematology & Oncology, University of Pittsburgh Medical Center, Pittsburgh, PA, United States; ^5^ Immunology Department, Egypt Center for Research and Regenerative Medicine (ECRRM), Cairo, Egypt; ^6^ Microbial Biotechnology Department, Biotechnology Research Institute, National Research Centre, Giza, Egypt

**Keywords:** HCC, antibody therapy, monoclonal antibodies, bispecific antibodies, antibody-drug conjugates, immune checkpoints inhibitors

## Abstract

Hepatocellular carcinoma (HCC) remains a leading cause of cancer-related deaths worldwide, underscoring the urgent need for innovative therapeutic strategies. Antibody-based therapies have emerged as a transformative approach, offering specificity and the potential to overcome the limitations of traditional treatments. This comprehensive review evaluates the current and emerging applications of antibody therapies in HCC, including monoclonal antibodies (mAbs), bispecific antibodies, and antibody-drug conjugates (ADCs). It explores their mechanisms of action, such as immune modulation, angiogenesis inhibition, and targeted cytotoxicity. Key advancements include the integration of immune checkpoint inhibitors (ICIs) like PD-1/PD-L1 and CTLA-4 inhibitors into clinical practice and the development of bispecific antibodies and ADCs targeting tumor-specific antigens like glypican-3. While these therapies have shown promise in improving patient outcomes, challenges such as tumor heterogeneity, resistance mechanisms, and immune-related adverse events persist. This review highlights recent clinical trial data, identifies areas for future research, and emphasizes the potential of combining antibody therapies with other modalities to enhance efficacy and overcome therapeutic barriers. By addressing these challenges and leveraging advancements in antibody engineering and biomarker discovery, antibody-based therapies hold significant promise for revolutionizing the treatment paradigm for HCC.

## 1 Overview of hepatocellular carcinoma

### 1.1 Epidemiology of HCC

HCC is the most common primary liver cancer, accounting for approximately 75-85% of all liver cancer cases. It is a major global health problem, with significant geographical variation in incidence rates due to differences in underlying risk factors and healthcare practices. HCC is the sixth most common cancer worldwide and the third leading cause of cancer-related deaths. In 2020, it was estimated that there were over 900,000 new cases and more than 830,000 deaths attributable to liver cancer globally (1). The highest incidence rates are found in East Asia and sub-Saharan Africa, with intermediate rates in Southern Europe and low rates in North America and Northern Europe (2).

East Asia Countries like China, Japan, and Korea have the highest incidence rates of HCC. In China alone, over 50% of the world’s HCC cases occur, largely due to the high prevalence of chronic hepatitis B virus (HBV) infection (3). Similar to East Asia, the high incidence in Sub-Saharan Africa is also linked to chronic HBV infection, which is often acquired perinatally or in early childhood (4). On the other hand, the incidence of HCC has been rising in Europe and North America, partly due to the increasing prevalence of hepatitis C virus (HCV) infection and metabolic dysfunction-associated steatotic liver disease (MASLD) (2).

Chronic HBV and HCV infections are the primary risk factors for HCC, responsible for about 80% of all cases globally (5). Regardless of the underlying cause, cirrhosis significantly increases the risk of developing HCC. Cirrhosis is most commonly caused by chronic viral hepatitis, alcoholic liver disease, and MASLD. Heavy alcohol consumption is a major risk factor, contributing to the development of cirrhosis and subsequently HCC (6). In some regions, such as sub-Saharan Africa and Southeast Asia, exposure to aflatoxin B1, a toxin produced by certain fungi in improperly stored grains and nuts, is a significant risk factor (7). Metabolic Disorders like obesity, diabetes, and MASLD are increasingly recognized as important risk factors, particularly in Western countries (8).

The global burden of HCC is expected to increase in the coming decades due to the aging population, the ongoing epidemic of metabolic risk factors, and variations in the success of HBV vaccination and HCV antiviral treatments. Efforts to control HCC must focus on prevention, early detection, and effective treatment of underlying liver diseases (9).

## 2 Drawbacks of traditional therapies

Traditional therapies for HCC have shown limited efficacy and considerable side effects, necessitating the development of innovative treatment strategies. While surgical resection and liver transplantation are considered potentially curative treatments for HCC, these options are viable only for a small subset of patients with early-stage disease and preserved liver function. Many patients are diagnosed at advanced stages, making them ineligible for surgery. Moreover, the availability of donor organs for transplantation is limited, and there is a risk of tumor recurrence even after surgery (10).

Treatments such as transarterial chemoembolization (TACE), radiofrequency ablation (RFA), and percutaneous ethanol injection (PEI) are commonly used for patients who are not candidates for surgery. While these therapies can control tumor growth and prolong survival, they are rarely curative and often associated with local recurrence (11). Additionally, their effectiveness can be limited in patients with large or multifocal tumors.

Systemic chemotherapy has historically shown limited efficacy in HCC, with low response rates and significant toxicity. The advent of targeted therapies, such as sorafenib and lenvatinib, has improved outcomes to some extent, but their benefits are modest, and they are often associated with adverse effects that can limit their use. Resistance to these therapies also develops over time, reducing their long-term effectiveness (12).

## 3 Antibody therapy: mechanisms of action in the HCC tumor microenvironment

Antibody-based therapies have transformed cancer treatment, including HCC, by targeting tumor-specific pathways, modulating the immune microenvironment, and delivering cytotoxic agents directly to cancer cells. The efficacy of these therapies is deeply influenced by the HCC TME, which is characterized by immune evasion, angiogenesis, and stromal interactions. Understanding these mechanisms provides insight into the rationale behind combination therapies, particularly those involving Immune-checkpoint Inhibitors (ICIs) and anti-angiogenic agents. The TME-centered approach to antibody therapy in HCC highlights the rationale for combination regimens. By disrupting angiogenesis, restoring immune surveillance, and selectively delivering cytotoxic agents, antibody-based therapies offer multi-faceted strategies to overcome resistance mechanisms in HCC. Future biomarker-driven approaches will further refine patient selection and enhance efficacy.

### 3.1 Targeting specific antigens in the TME

Monoclonal antibodies (mAbs) exert anti-tumor effects by selectively binding tumor-associated antigens, disrupting key oncogenic pathways, and engaging immune effector cells.

#### 3.1.1 Anti-VEGF therapy and its role in HCC

Vascular endothelial growth factor (VEGF) is a key driver of angiogenesis in the HCC TME, promoting neovascularization, immune suppression, and tumor progression. Bevacizumab, an anti-VEGF monoclonal antibody, inhibits VEGF-A, leading to vascular normalization, improved immune infiltration, and reduced tumor hypoxia (13). Anti-VEGF therapy complements ICIs such as atezolizumab, nivolumab, and pembrolizumab. By enhancing T-cell infiltration, VEGF-induced abnormal vasculature limits immune cell access to the tumor. Bevacizumab normalizes blood vessels, allowing better T-cell penetration (14).

Anti-VEGF antibodies like bevacizumab enhance the effectiveness of PD-1/PD-L1 inhibitors in hepatocellular carcinoma (HCC) through multiple mechanisms. By inhibiting angiogenesis, bevacizumab limits the formation of tumor-associated blood vessels, thereby increasing T-cell infiltration while reducing the presence of immunosuppressive cells within the TME (15, 16). This shift fosters conditions that promote immune activation and improve the response to PD-1/PD-L1 blockade (17). Additionally, bevacizumab contributes to vascular normalization, which optimizes oxygenation and facilitates the efficient delivery of therapeutic agents, further enhancing immune responses (18). Moreover, by alleviating tumor hypoxia, it influences PD-L1 expression, creating a more pro-inflammatory environment that makes tumor cells more vulnerable to immune-mediated destruction (19). These combined effects support the rationale for using anti-VEGF antibodies alongside PD-1/PD-L1 inhibitors to improve treatment outcomes in HCC.

#### 3.1.2 Antibody-dependent cellular cytotoxicity

Monoclonal antibodies can engage innate immune responses through Fcγ receptor (FcγR)-mediated ADCC, in which antibody-coated tumor cells are recognized and destroyed by natural killer (NK) cells and macrophages (20). For example, anti-GPC3 antibodies, which target glypican-3 (GPC3), a cell surface glycoprotein overexpressed in HCC, can induce ADCC, leading to tumor cell lysis (21).

### 3.2 Immune modulation and checkpoint blockade in HCC

HCC tumors create an immunosuppressive microenvironment dominated by exhausted T cells, myeloid-derived suppressor cells (MDSCs), and Tregs, which collectively inhibit anti-tumor immunity (22).

#### 3.2.1 PD-1/PD-L1 axis: reversing t-cell exhaustion

PD-1 (on T cells) binds PD-L1 (on tumor or immune cells), suppressing T-cell activation and proliferation. Nivolumab and pembrolizumab restore T-cell function by blocking PD-1/PD-L1 interaction, reinvigorating exhausted CD8+ T cells (17). CTLA-4 blockade (e.g., Ipilimumab, Tremelimumab) acts earlier in the immune response by expanding effector T cells and reducing regulatory T cells (Tregs) (23). PD-1 blockade acts later, preventing T-cell exhaustion within the TME. CTLA-4 blockade enhances priming and expansion of tumor-reactive T cells. PD-1 blockade sustains the activity of these expanded T cells in the TME. This synergistic mechanism is demonstrated in STRIDE (Tremelimumab + Durvalumab) from the HIMALAYA trial, which showed OS benefit in HCC (24).

Emerging treatment modalities include TIGIT inhibitors (e.g., Tiragolumab) targeting TIGIT which is an alternative checkpoint that suppresses NK and CD8+ T cells; blocking TIGIT can synergize with PD-1 blockade (25). LAG-3 inhibitors (e.g., Relatlimab): LAG-3 restrains exhausted T cells; LAG-3 blockade enhances anti-PD-1 efficacy (26).

### 3.3 Antibody-drug conjugates for targeted cytotoxicity

In this approach, ADCs deliver potent chemotherapy directly to tumor cells, minimizing off-target toxicity where ADC binds to the tumor antigen leading to internalization of the cytotoxic compound by the tumor cell. Cytotoxic payload (e.g., microtubule inhibitor) is released intracellularly hence inducing apoptosis (27).

### 3.4 Bispecific antibodies: dual-targeting strategy

BsAbs bridges T cells and tumor cells, enhancing immune cell cytotoxicity (28). For example, Blinatumomab (CD19 x CD3) in leukemia; GPC3 x CD3 BsAbs are being explored for HCC (29). Mechanistically BsAbs improve specificity while reducing systemic toxicity compared to ICIs (30).

## 4 Monoclonal antibodies in HCC treatment

Monoclonal antibodies represent a significant advancement in cancer treatment. These therapies are designed to specifically target antigens expressed on cancer cells, thereby reducing off-target effects and enhancing therapeutic efficacy. In HCC, mAbs such as bevacizumab, which targets VEGF, have shown promising results, particularly when used in combination with other treatments like atezolizumab, an anti-programmed cell death protein 1 (PD-1) antibody (anti-PD-L1) (31) (32).

Below is an in-depth exploration of key monoclonal antibodies used in HCC treatment.

### 4.1 Overview of monoclonal antibodies

mAbs are laboratory-generated molecules engineered to serve as substitute tools that can restore, enhance, or mimic the attack of the human immune system on cancer cells. They are highly specific, targeting particular antigens associated with cancer cells, and can work through various mechanisms, including blocking growth signals, inducing apoptosis, and recruiting immune cells to attack tumors (33).

Key mAbs in HCC treatment include:

### 4.2 mAbs targeting angiogenesis

#### 4.2.1 Bevacizumab (Avastin)

It is a monoclonal antibody that targets and inhibits VEGF, a key molecule involved in angiogenesis (the formation of new blood vessels). By inhibiting VEGF, bevacizumab reduces the blood supply to tumors, which is essential for their growth and metastasis (34). The combination of bevacizumab with atezolizumab (an anti-PD-L1 antibody) has shown promising results in the treatment of unresectable HCC. The IMbrave150 trial was a global, randomized, open-label, phase III study evaluating the efficacy and safety of atezolizumab combined with bevacizumab versus sorafenib as first-line treatment for patients with unresectable hepatocellular carcinoma (HCC). Patients were randomized in a 2:1 ratio to receive either the combination therapy or sorafenib (35).

At the primary analysis, with a median follow-up of 8.6 months, the combination therapy demonstrated a statistically significant improvement in (OS) compared to sorafenib. The hazard ratio (HR) for death was 0.58 (95% CI: 0.42, 0.79; p < 0.001), indicating a 42% reduction in the risk of death. The median progression-free survival (PFS) was 6.8 months for the combination therapy versus 4.3 months for sorafenib, with an HR of 0.59 (95% CI: 0.47, 0.76; p < 0.001). The objective response rate (ORR) was 27% for the combination therapy compared to 12% for sorafenib (36).

An updated analysis with an additional 12 months of follow-up (median follow-up of 15.6 months) confirmed the sustained benefit of the combination therapy. The median OS was 19.2 months for the combination therapy versus 13.4 months for sorafenib, with an HR of 0.66 (95% CI: 0.52, 0.85; p = 0.0009). The median PFS was 6.9 months for the combination therapy versus 4.3 months for sorafenib, with an HR of 0.65 (95% CI: 0.53, 0.81; p = 0.0001). The ORR was 30% for the combination therapy compared to 11% for sorafenib (37). These results established atezolizumab plus bevacizumab as a new standard of care for patients with unresectable HCC, offering significant improvements in survival outcomes over sorafenib.

#### 4.2.2 Ramucirumab (Cyramza)

It is a monoclonal antibody that targets VEGF receptor-2 (VEGFR-2), thereby inhibiting the VEGF signaling pathway involved in tumor angiogenesis. By blocking VEGFR-2, ramucirumab helps to reduce the growth of blood vessels that supply the tumor (38). Ramucirumab has shown efficacy in patients with advanced HCC, particularly in those with elevated alpha-fetoprotein (AFP) levels. The REACH-2 trial was a randomized, double-blind, placebo-controlled, phase III study evaluating the efficacy and safety of ramucirumab as a second-line treatment for patients with advanced hepatocellular carcinoma (HCC) and elevated alpha-fetoprotein (AFP) levels (≥400 ng/mL) who had previously been treated with sorafenib (39). In this study, 292 patients were randomized in a 2:1 ratio to receive either ramucirumab (8 mg/kg intravenously every two weeks) or placebo. The primary endpoint was OS, with secondary endpoints including PFS and ORR.

The results demonstrated a statistically significant improvement in OS for patients receiving ramucirumab compared to placebo. The median OS was 8.5 months for the ramucirumab group versus 7.3 months for the placebo group, with a hazard ratio (HR) of 0.71 (95% CI: 0.531–0.949; p = 0.0199). The median PFS was 2.8 months for the ramucirumab group compared to 1.6 months for the placebo group, with an HR of 0.452 (95% CI: 0.339–0.603; p < 0.0001). The ORR was 4.6% for the ramucirumab group versus 1.1% for the placebo group (40).

### 4.3 mAbs targeting immune checkpoints

Programmed cell death protein 1 (PD-1) is an inhibitory receptor expressed on T cells, and its ligand, PD-L1, can be expressed on tumor cells and other cells within the TME. The interaction between PD-1 and PD-L1 inhibits T-cell activity, reducing the immune response against the tumor (41). Cytotoxic T-lymphocyte-associated protein 4 (CTLA-4) is another inhibitory receptor found on T cells. It competes with the costimulatory receptor CD28 for binding to B7 molecules (CD80/CD86) on antigen-presenting cells, thereby attenuating T-cell activation early in the immune response (23).

**Figure 1:**
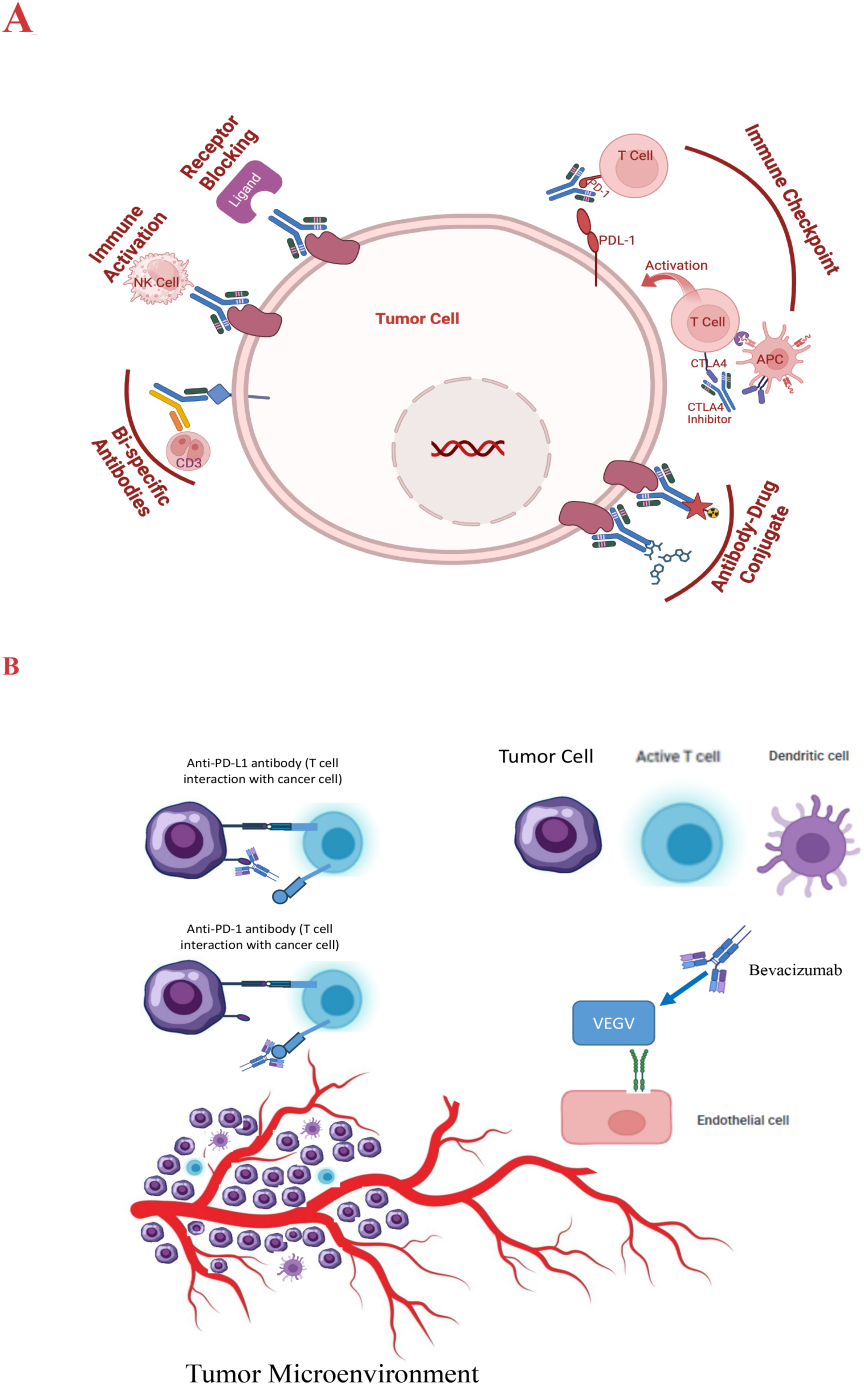
**(A)** Schematic presentation of the modes of action of antibody therapy of HCC. **(B)** VEGF signaling inhibition pathway and the impact on the tumor microenvironment.

ICIs are monoclonal antibodies designed to block these inhibitory pathways, enhancing the immune system’s ability to recognize and destroy cancer cells (42). ICIs, such as PD-1/Programmed death ligand 1 (PD-L1) and Cytotoxic T-lymphocyte-associated protein 4 (CTLA-4), are often exploited by cancer cells to evade immune detection. ICIs, like nivolumab and pembrolizumab, have demonstrated efficacy in a subset of HCC patients, leading to durable responses and improved survival in some cases (43). **Figure 1** describes a schematic representation of the types and modes of action of antibody-based therapy of HCC.

Pembrolizumab is a humanized IgG4 monoclonal antibody that also targets PD-1, preventing it from binding to PD-L1 and PD-L2, thus enhancing T-cell activity against tumor cells (44). The KEYNOTE-240 trial evaluated pembrolizumab in patients with advanced HCC who had previously been treated with sorafenib (45). While the trial did not meet its primary endpoints of OS and PFS, pembrolizumab demonstrated a clinically meaningful improvement in both measures. The ORR was 18.3%, and some patients experienced prolonged responses (46). Pembrolizumab is approved for the treatment of HCC following sorafenib based on the results from the KEYNOTE-224 (47) and KEYNOTE-240 trials (46).

The combination of anti-VEGF therapy (e.g., bevacizumab) with immune ICIs such as atezolizumab or pembrolizumab is not merely additive but mechanistically synergistic, as it targets distinct but interconnected pathways within the TME. The efficacy of PD-1/PD-L1 inhibitors depends on adequate T-cell priming, activation, infiltration, and persistence—all of which are negatively impacted by VEGF signaling. Bevacizumab enhances ICI efficacy by overcoming VEGF-mediated immunosuppression at multiple levels:

#### 4.3.1 Reversing VEGF-induced immune suppression

VEGF inhibits dendritic cell (DC) maturation, leading to poor antigen presentation and impaired T-cell priming (48). Bevacizumab restores DC function, thereby enhancing tumor antigen presentation and T-cell activation (14).

#### 4.3.2 Enhancing T-cell infiltration by normalizing tumor vasculature

Pathological angiogenesis induced by VEGF results in chaotic, leaky blood vessels, limiting effective immune cell infiltration (49). Anti-VEGF therapy promotes vascular normalization, stabilizing endothelial junctions and pericyte coverage, allowing efficient CD8+ T-cell entry into tumors (50). This effect reduces hypoxia, which in turn lowers immunosuppressive regulatory T cells (Tregs) and myeloid-derived suppressor cells (MDSCs) (51).

#### 4.3.3 Upregulating PD-L1 expression to enhance ICI sensitivity

VEGF-induced hypoxia upregulates PD-L1 expression on tumor cells, promoting immune evasion (52). Bevacizumab reduces hypoxia, downregulating PD-L1 expression and making tumor cells more susceptible to PD-1/PD-L1 blockade (53).

#### 4.3.4 Increasing CD8+ T-cell cytotoxicity and IFN-γ release

VEGF suppresses effector T-cell function via multiple mechanisms, including induction of exhaustion markers (54). Bevacizumab reverses this suppression, enhancing interferon-gamma (IFN-γ) production and cytotoxic activity of CD8+ T cells (55).

The clinical evidence supporting this synergy was evident by the IMbrave150 trial (Atezolizumab + Bevacizumab) which demonstrated that this combination achieved superior OS and PFS compared to sorafenib, confirming the mechanistic synergy in HCC (36). Unlike single-agent ICIs, which are often ineffective in highly immunosuppressive tumors, combining anti-VEGF therapy with PD-1/PD-L1 blockade overcomes multiple resistance mechanisms in the TME. This approach enhances antigen presentation, T-cell infiltration, immune activation, and cytotoxicity, making it a cornerstone of modern HCC therapy.

Identifying biomarkers that predict response to ICIs is crucial for optimizing patient selection and improving outcomes. Potential biomarkers include PD-L1 expression, tumor mutational burden (TMB), and specific gene signatures associated with immune response (56).

**Figure 2:**
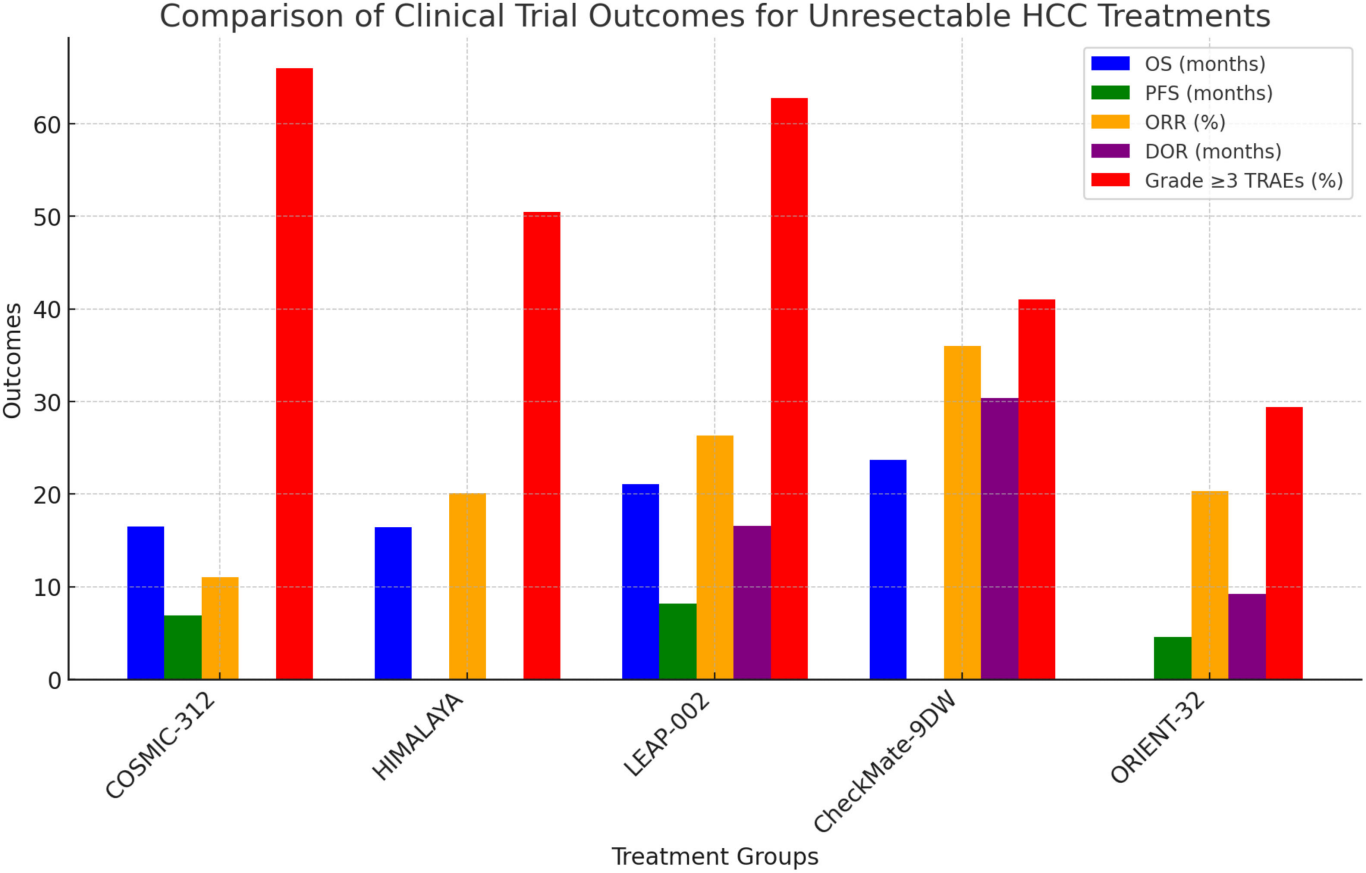
Comparison of clinical trial outcomes for unresectable HCC treatments. Confidence Progression-Free Survival (PFS), Objective Response Rate (ORR), Overall Survival (OS).

Ongoing research focuses on combining ICIs with other treatments, such as targeted therapies, locoregional treatments, and other immunotherapies, to enhance their efficacy and overcome resistance. Understanding the optimal sequencing and combination of these therapies is critical for maximizing their benefits (57) (**Figure 2**).

The dual-targeting capability of bispecific antibodies allows them to bring two different cells or molecules into proximity, thereby enhancing their therapeutic efficacy (58). One of the primary mechanisms by which bispecific antibodies function is by bringing T cells, which express CD3, into proximity with cancer cells expressing a specific tumor antigen. This engagement can lead to T cell activation, proliferation, and subsequent killing of the cancer cells. For example, blinatumomab, a bispecific T cell engager (BiTE), targets CD19 on B cells and CD3 on T cells, facilitating T cell-mediated lysis of B cell malignancies (59).

While bispecific antibodies are well established in the treatment of hematologic malignancies, their application in solid tumors, including HCC, is still in the early stages of research. Preclinical and early clinical trials are investigating the efficacy and safety of various bispecific constructs in HCC (60). Researchers are developing bispecific antibodies targeting specific antigens expressed on HCC cells, such as glypican-3 (GPC3). For instance, a bispecific antibody targeting GPC3 and CD3 is designed to redirect T cells to GPC3-expressing HCC cells, thereby promoting targeted immune responses against the tumor (29).

Some bispecific antibodies are designed to combine the mechanisms of immune checkpoint inhibition and T-cell engagement. These constructs can block inhibitory signals while simultaneously directing T cells to the tumor, enhancing the overall immune response (61). One of the significant challenges in treating solid tumors like HCC with bispecific antibodies is the complex TME. Factors such as immunosuppressive cells, physical barriers, and cytokines within the tumor milieu can hinder the efficacy of bispecific antibodies. The engagement of immune cells, especially T cells, must be tightly regulated to avoid excessive immune activation and potential off-target effects, which could lead to adverse events such as cytokine release syndrome (CRS) (62). Ongoing research aims to improve the specificity and potency of bispecific antibodies. Strategies include optimizing the binding affinities to the target antigens and engineering the antibody structures to enhance their stability and efficacy.

Several early-phase clinical trials are exploring the safety and efficacy of bispecific antibodies in patients with advanced HCC. These trials are essential for understanding the pharmacokinetics, optimal dosing, and potential therapeutic benefits of these novel agents. A phase II clinical trial was performed to investigate the efficacy of AK104 plus lenvatinib in patients with unresectable HCC, BCLC stage B or C, Child-Pugh class A, who had not previously received systemic treatment (63). AK104 is a humanized IgG1 bispecific antibody that simultaneously binds to PD-1 and CTLA-4. This single-arm, multicenter trial involved 30 patients who received AK104 intravenously every two or three weeks alongside daily oral lenvatinib. The primary endpoint was the objective response rate (ORR) per RECIST v1.1 criteria. Secondary endpoints included disease control rate (DCR), duration of response (DOR), PFS, and OS. As of February 1, 2021, among 18 evaluable patients, the study reported an ORR of 44.4% and a DCR of 77.8%. The median PFS had not been reached at the time of reporting. Treatment-related adverse events (TRAEs) occurred in 83.3% of patients, with Grade 3 TRAEs in 26.7%. No Grade 4 TRAEs or treatment-related deaths were observed. Common TRAEs included increased AST and ALT levels, decreased platelet and neutrophil counts, and increased blood bilirubin, predominantly of Grade 1 or 2 severity. Further studies with longer follow-up are needed to assess the durability of the response.

The success of bispecific antibodies in other cancers provides a strong rationale for their development in HCC. Future research will likely focus on combining bispecific antibodies with other therapeutic modalities, such as ICIs, tyrosine kinase inhibitors, and locoregional therapies, to enhance their efficacy and overcome resistance mechanisms.

### 4.4 Emerging monoclonal antibodies

Atezolizumab and durvalumab are mAb designed to target and inhibit the activity of the programmed death-ligand 1 (PD-L1) protein, a critical component in immune regulation and cancer immune evasion (64). They specifically bind to PD-L1 on tumor cells and antigen-presenting cells. Under normal conditions, PD-L1 binds to PD-1 receptors on T cells, inhibiting T cell activity and allowing cancer cells to evade immune detection. By blocking the interaction between PD-L1 and PD-1, both atezolizumab and durvalumab prevent the “off” signal from being sent to T cells. This blockade helps restore T cell activity, enabling the immune system to recognize and attack cancer cells more effectively. Both mAbs have been extensively investigated for HCC treatment either alone or in combination with each other or with other mAbs, as discussed earlier.

Research continues to identify and develop new monoclonal antibodies for treating HCC. Several novel targets are under investigation, including:

Glypican-3 (GPC3): GPC3 is a cell surface protein that is overexpressed in HCC. Monoclonal antibodies targeting GPC3 are being developed to exploit this specificity (21).

C-MET: The hepatocyte growth factor receptor (c-MET) is implicated in HCC progression. Antibodies targeting c-MET are being studied for their potential to inhibit tumor growth and metastasis (65).

Additionally, ongoing studies are exploring combinations of monoclonal antibodies with other treatment modalities, such as tyrosine kinase inhibitors, chemotherapy, and locoregional therapies, to enhance efficacy and overcome resistance mechanisms.

T-cell immunoreceptor with Ig and ITIM domains (TIGIT) has emerged as a promising target in hepatocellular carcinoma (HCC) immunotherapy. TIGIT is an immune checkpoint receptor that, when inhibited, can enhance T-cell and natural killer (NK) cell responses against tumors (66). Recent clinical trials have explored the efficacy of combining anti-TIGIT antibodies with existing therapies in HCC. A notable study is the MORPHEUS-liver trial, a phase Ib/II randomized trial evaluating the addition of tiragolumab, an anti-TIGIT monoclonal antibody, to the standard regimen of atezolizumab (an anti-PD-L1 antibody) and bevacizumab (an anti-VEGF antibody) in patients with unresectable or metastatic HCC. The trial reported a confirmed objective response rate of 43% in the tiragolumab combination group, compared to 11% in the control group receiving only atezolizumab and bevacizumab. Median progression-free survival was also extended to 12.3 months in the tiragolumab group versus 4.2 months in the control group. Importantly, the addition of tiragolumab did not result in a substantial increase in treatment-related adverse events, suggesting a favorable safety profile (67).

The phase III IMbrave152/SKYSCRAPER-14 trial aimed to assess the efficacy and safety of combining tiragolumab with atezolizumab and bevacizumab as a first-line treatment for patients with advanced HCC. This randomized, double-blind, placebo-controlled study aims to determine whether the addition of tiragolumab can improve OS and PFS compared to the standard therapy alone (68). These studies underscore the potential of targeting TIGIT in combination with established immunotherapies to enhance anti-tumor responses in HCC. Ongoing and future trials will provide more definitive insights into the clinical benefits of this approach.

## 5 Antibody-drug conjugates

ADCs consist of three main components: a monoclonal antibody specific to a tumor-associated antigen, a potent cytotoxic drug, and a linker that connects the drug to the antibody. Upon binding to its target antigen on the cancer cell surface, the ADC-antigen complex is internalized into the cell via endocytosis (69). Once inside the cancer cell, the ADC is trafficked to lysosomes where the linker is cleaved, releasing the cytotoxic drug. The released drug then exerts its cytotoxic effects, typically by disrupting critical cellular processes such as DNA replication or microtubule function, leading to cell death. The primary advantage of ADCs is their ability to deliver high concentrations of cytotoxic drugs directly to cancer cells, enhancing anti-tumor efficacy while reducing systemic exposure and associated toxicities. This targeted approach is particularly beneficial for cancers with specific and well-characterized surface antigens.

Glypican-3 (GPC3) is a cell surface protein overexpressed in HCC but not in normal adult tissues, making it an attractive target for ADC development. Several GPC3-targeting ADCs are under investigation, including codrituzumab (also known as GC33), which is linked to a cytotoxic drug and designed to target GPC3-expressing HCC cells (70). Preclinical studies have demonstrated that GPC3-targeting ADCs can effectively bind to HCC cells, induce internalization, and deliver cytotoxic payloads, resulting in significant anti-tumor activity *in vitro* and *in vivo* (71). In an imaging study, each patient received an intravenous injection of approximately 185 MBq (10 mg) of I-124 codrituzumab. Serial positron emission tomography/computed tomography (PET/CT) scans were conducted over seven days to assess the biodistribution and tumor uptake of the radiolabeled antibody. Pharmacokinetic analyses were performed using blood samples collected at specified intervals. Seven patients, undergoing treatment with sorafenib and cold codrituzumab (2.5 or 5 mg/kg), had repeat imaging with co-infusion of I-124 codrituzumab. Three patients who progressed on sorafenib/immunotherapy were re-imaged after a four-week washout period to assess antigen presence. Thirteen out of fourteen patients exhibited tumor localization of I-124 codrituzumab, with noted heterogeneity in tumor uptake. The pharmacokinetic profile of I-124 codrituzumab was comparable to that of other intact iodinated humanized IgG antibodies. No significant adverse events related to I-124 codrituzumab were observed during the study period. The study concluded that I-124 codrituzumab effectively localized to tumors in most HCC patients, demonstrating a favorable pharmacokinetic profile and safety. These findings suggest the potential utility of I-124 codrituzumab in imaging applications for HCC, warranting further investigation.

Despite their potential, ADCs face several challenges. The development of resistance through antigen downregulation or modifications in intracellular trafficking pathways can reduce efficacy. Additionally, the heterogeneity of antigen expression within tumors can limit the effectiveness of ADCs. The stability of the linker and the choice of the cytotoxic drug also play critical roles in the overall success of ADCs.

Multiple clinical trials are evaluating the safety and efficacy of ADCs in patients with HCC. These trials aim to determine optimal dosing, assess therapeutic outcomes, and identify potential biomarkers for response. Early-phase clinical trials have shown promising results for ADCs targeting GPC3 in HCC. For instance, in a Phase Ib, open-label, dose-escalation study (72), 41 patients with advanced HCC, aged ≥18 years, ECOG performance status 0–1, Child-Pugh class A or B7, adequate organ function, and no prior systemic therapy were enrolled. Patients received intravenous codrituzumab at varying doses (2.5 mg/kg weekly, 5 mg/kg weekly, 10 mg/kg weekly, 1600 mg every two weeks, or 1600 mg weekly) in combination with oral sorafenib 400 mg twice daily. No patients achieved a complete or partial ORR and 9 patients (25.7%) experienced stable disease as their best response. The majority of patients exhibited disease progression. Two cases encountered Dose-Limiting Toxicities (DLTs): one case of grade 3 hyponatremia at the 5 mg/kg dose and one case of grade 3 hyponatremia and hyperglycemia at the 1600 mg every two weeks dose. 80% of patients experienced treatment-related adverse events (AEs), with the most common being increased AST in 10 patients (25%), increased ALT in 3 patients (7.5%), and increased lipase in 10 patients (25%). Most AEs were grade 1 or 2; however, some patients experienced grade 3 elevations in liver enzymes and lipase. The maximum concentration (C_max) and area under the curve (AUC) of codrituzumab and sorafenib were comparable to those observed in single-agent studies, indicating no significant drug-drug interactions. The study concluded that the combination of codrituzumab and sorafenib was generally well-tolerated at the tested doses, with manageable safety profiles. However, the lack of objective responses indicates limited efficacy in this patient population. The study suggests that while codrituzumab effectively targets GPC3-expressing tumors, its combination with sorafenib does not provide significant clinical benefit in advanced HCC.

Combining ADCs with other treatment modalities, such as immune ICIs, tyrosine kinase inhibitors, or locoregional therapies, may enhance therapeutic efficacy and overcome resistance mechanisms.

## 6 Challenges in antibody therapy for HCC

While antibody therapies, including mAbs, bispecific antibodies, and antibody-drug conjugates (ADCs), have shown significant promise in the treatment of hepatocellular carcinoma (HCC), several challenges hinder their optimal effectiveness. Understanding and addressing these challenges is crucial for improving patient outcomes.

One of the significant challenges in antibody therapy is the development of resistance, both primary (innate) and acquired. Primary resistance occurs when patients do not respond to therapy from the outset, while acquired resistance develops after an initial period of responsiveness. Mechanisms of resistance include antigen loss or modification, changes in intracellular signaling pathways, and adaptive immune resistance (73).

Tumor cells can downregulate or lose the expression of target antigens, rendering antibody therapies ineffective. For example, in the context of immune checkpoint inhibitors, tumors may downregulate PD-L1 or mutate the PD-1/PD-L1 pathway components to escape immune detection (74). Tumor cells can also activate alternative signaling pathways to bypass the inhibited pathway. For instance, resistance to anti-VEGF therapy like bevacizumab can arise through the activation of alternative angiogenic pathways (75).

Another mechanism by which tumors can evade antibody therapy is by creating an immunosuppressive microenvironment by recruiting regulatory T cells (Tregs), myeloid-derived suppressor cells (MDSCs), and secreting immunosuppressive cytokines, which can inhibit the effectiveness of immune-modulating antibody therapies (76, 77).

One of the challenges encountered in antibody therapy is the Immune-Related Adverse Events (irAEs). Antibody therapies, particularly immune checkpoint inhibitors, can cause irAEs due to heightened immune activity. These adverse effects can affect various organs and systems, leading to conditions such as colitis, hepatitis, pneumonitis, dermatitis, and endocrinopathies (78). Managing irAEs often requires immunosuppressive treatment, which can complicate therapy and impact patient quality of life.

ADCs and bispecific antibodies, while designed to be highly specific, can sometimes bind to antigens expressed at low levels on normal tissues, leading to On-Target, Off-Tumor Toxicity. This can result in adverse effects such as myelosuppression, hepatotoxicity, and nephrotoxicity (79).

While ICIs have shown significant promise in the treatment of HCC, several challenges remain. Some patients do not respond to ICIs (primary resistance), and others who initially respond may eventually develop resistance (acquired resistance). Mechanisms of resistance include upregulation of alternative immune checkpoints, loss of antigen presentation, and immunosuppressive TME (80). ICIs can cause a range of Immune-Related Adverse Events (irAEs) due to increased immune activity. Common irAEs include colitis, hepatitis, dermatitis, and endocrinopathies. Managing these side effects requires careful monitoring and prompt intervention with immunosuppressive therapies when necessary (81).

Nivolumab is a human IgG4 monoclonal antibody that targets PD-1, blocking its interaction with PD-L1 and PD-L2. This blockade enhances T-cell responses against tumor cells (82). The CheckMate 459 trial was a phase III study comparing nivolumab to sorafenib as first-line treatments for advanced hepatocellular carcinoma (HCC). The primary endpoint was OS. Results failed to show a statistically significant difference between the outcomes of the two treatments (a median OS of 16.4 months for nivolumab and 14.7 months for sorafenib). This was followed by the setup of the phase 3 CheckMate-9DW trial to evaluate the efficacy and safety of the combination of nivolumab plus ipilimumab as a first-line treatment for patients with advanced, unresectable hepatocellular carcinoma (HCC) (83). The trial compares this immunotherapy regimen against the current standard-of-care treatments, such as sorafenib or lenvatinib. Key endpoints include OS, PFS, and ORR, with a particular focus on whether the combination can deliver a significant survival benefit while maintaining a manageable safety profile. Preliminary findings have been promising enough to support further regulatory submissions, including a supplemental Biologics License Application (sBLA) for first-line treatment in advanced HCC. This underscores the importance of combination therapy in cases where monotherapy fails to provide an efficient therapeutic option.

The development, production, and administration of antibody therapies are expensive, making them costly for healthcare systems and patients. This high cost can limit accessibility, particularly in low- and middle-income countries. The economic burden of these therapies is a significant barrier to their widespread use (84). Another challenge is that administering antibody therapies often requires specialized infrastructure and expertise. This includes facilities for intravenous infusions, monitoring for adverse effects, and managing complications. Ensuring a reliable supply chain for biological medications can be challenging because of logistical obstacles, such as storage and transportation needs. Another challenge for the widespread use of antibody therapies is the lack of early screening programs for tumor detection which allows for optimal selection of therapy and better response (85). Approval and regulation of novel therapies can be intricate and differ greatly among countries, resulting in delays in accessing new treatments (86). In regions with limited healthcare infrastructure, the delivery of these advanced therapies can be challenging. Insufficient local clinical trials and research on HCC in LMICs may lead to a lack of information regarding the efficacy of these therapies in different populations (87).

The TME in HCC is highly immunosuppressive, characterized by the presence of Tregs, MDSCs, and immunosuppressive cytokines like TGF-β and IL-10. This environment can inhibit the activity of therapeutic antibodies, particularly those designed to stimulate an anti-tumor immune response (88). The TME and tumor cells themselves can be highly heterogeneous, meaning that different areas of the tumor may respond differently to therapy. This heterogeneity can lead to incomplete responses and relapse (89).

Large molecules like antibodies often have difficulty penetrating solid tumors effectively due to their size and the dense extracellular matrix of tumors. This can result in suboptimal drug delivery to all areas of the tumor (90). The stability and half-life of antibodies in the bloodstream can affect their efficacy. Some antibodies may be rapidly cleared from the body or degraded, reducing their therapeutic potential (91).

### 6.1 Biomarker-based patient selection

HCC is a highly heterogeneous disease with various etiologies, including hepatitis B or C infection, alcohol-related liver disease, and non-alcoholic steatohepatitis. This biological complexity makes identifying universal biomarkers predicting response to antibody-based therapies challenging (92). Given the variability in TMEs, genetic mutations, and immune profiles, stratifying patients using predictive biomarkers is essential for optimizing therapeutic efficacy and minimizing unnecessary exposure to ineffective treatments (93). Key Biomarkers for Antibody Therapy Response in HCC include:

1. PD-L1 Combined Positive Score (CPS) for Immune Checkpoint Inhibitors: Programmed death-ligand 1 (PD-L1) expression has been widely investigated as a potential biomarker for response to ICIs like nivolumab and pembrolizumab. Studies have suggested that a higher PD-L1 combined positive score (CPS), which accounts for PD-L1 expression in tumor and immune cells, correlates with better responses to anti-PD-1/PD-L1 therapy (94). However, PD-L1 expression alone has not been a definitive predictor in HCC, as responses to ICIs have also been observed in patients with low or undetectable PD-L1 levels. This highlights the need for additional biomarkers or combination approaches to refine patient selection.
2. Tumor Mutational Burden (TMB) is a measure of the number of somatic mutations within a tumor and has been explored as a potential predictor of response to immunotherapy (95). While higher TMB has been associated with improved responses to ICIs in various cancers (e.g., melanoma, lung cancer), its role in HCC remains less well-defined. Emerging evidence suggests that a subset of HCC patients with high TMB may derive greater benefit from checkpoint blockade, but further studies are needed to validate this as a robust biomarker in liver cancer.
3. Glypican-3 (GPC3) Expression for Targeted Antibody Therapies: GPC3 has been targeted for antibody-based therapies, including antibody-drug conjugates (ADCs) and bispecific T-cell engagers (BiTEs) (96). Biomarker-driven patient selection based on GPC3 expression could enhance the efficacy of these novel therapies, making it a promising avenue for future personalized treatment strategies (97).
4. Alpha-fetoprotein (AFP) is a well-established serum biomarker in HCC and has been explored as a predictive marker for treatment response. The REACH-2 trial demonstrated that patients with AFP levels ≥400 ng/mL derived significant survival benefits from ramucirumab, a VEGFR-2 monoclonal antibody (98). This finding led to FDA approval of ramucirumab for HCC patients with high AFP levels, establishing AFP as the first biomarker-driven selection criterion for an HCC therapy.

Despite these advancements, significant challenges remain in identifying and validating reliable biomarkers for antibody therapy in HCC. The different etiologies (HBV, HCV, alcohol, NAFLD) influence tumor biology and immune responses, complicating the development of a one-size-fits-all biomarker. Here arises the need for dynamic biomarkers such as PD-L1 whose expression may change over time due to treatment-induced immune modulation, requiring longitudinal monitoring. Multimodal biomarker approaches combining genomic (TMB, GPC3), proteomic (AFP, PD-L1), and immunological markers may enhance the predictive power for treatment response.

### 6.2 Immune-related adverse events in HCC treatment

Although rare, immune myocarditis is a serious and potentially fatal immune-related adverse event (irAE) associated with ICIs, particularly in combination regimens. Immune myocarditis is thought to result from T-cell infiltration and immune-mediated destruction of cardiac myocytes, leading to impaired cardiac function. The incidence of immune myocarditis in ICI-treated patients is estimated to be 0.1–0.3%, but it carries a high mortality rate of 40–50%, making early detection and aggressive management essential (99).

Timely identification of immune myocarditis can significantly improve outcomes. Key strategies include routine measurement of cardiac troponins (e.g., hs-TnI or hs-TnT), which can detect subclinical myocarditis before overt cardiac dysfunction develops. Another approach is the Electrocardiogram (ECG) and Echocardiography, where abnormalities (ST-segment changes, conduction delays) and echocardiographic findings (reduced ejection fraction, regional wall motion abnormalities) may indicate myocarditis. Cardiac Magnetic Resonance Imaging (MRI) with late gadolinium enhancement on MRI can help confirm myocarditis in ambiguous cases (100).

These irAEs can be managed by immediate administration of High-dose corticosteroids (methylprednisolone), which should be initiated upon suspicion of immune myocarditis, with a slow taper over weeks to prevent relapse. Immunosuppressive Therapy using Abatacept, a CTLA-4 agonist, due to its ability to dampen T-cell activation while preserving anti-tumor immunity. Infliximab is generally avoided due to its potential to exacerbate cardiac inflammation. A Multidisciplinary Approach with Cardio-oncology collaboration is critical for optimizing treatment decisions and monitoring for long-term sequelae (101).

## 7 Future directions and innovations

The future of antibody therapies for HCC involves the development of next-generation antibodies designed to improve efficacy, reduce resistance, and minimize side effects. These innovations aim to address the current limitations of existing therapies and offer new hope for patients with advanced HCC.

Smaller antibody fragments and nanobodies (single-domain antibodies) are being developed to improve tissue penetration and reduce immunogenicity. These smaller molecules can access tumor sites more effectively than full-sized antibodies, potentially enhancing therapeutic outcomes (102). Advances in antibody engineering have led to the development of bispecific and multispecific antibodies that can simultaneously target multiple antigens or pathways. This approach can enhance the specificity and potency of the immune response against cancer cells, reducing the likelihood of resistance and improving overall efficacy (103).

Ongoing research is focused on discovering new tumor-specific antigens that antibody therapies can target. Glypican-3 (GPC3) is an example of a promising target in HCC, and further identification of such targets can lead to the development of more effective treatments (104). **Table 1** summarizes the outcome of the current guideline studies vs the exploratory studies for the immunotherapeutic regimens for HCC.

**Table 1:** Guideline vs. Exploratory Regimens in HCC.

Regimen	Category	Key trials	Primary outcomes
Atezolizumab + Bevacizumab (IMbrave150)	Guideline-Recommended	IMbrave150	OS: 19.2m vs 13.4m (HR: 0.66, P<0.001)
Durvalumab + Tremelimumab (STRIDE)	Guideline-Recommended	HIMALAYA	OS: 16.4m vs 13.8m (HR: 0.78, P=0.0035)
Lenvatinib + Pembrolizumab	Exploratory	LEAP-002	OS: 21.1m vs 19.0m (HR: 0.836)
AK104 + Lenvatinib	Exploratory	NCT05020236	ORR: 34.8% | DCR: 78.3%
TIGIT Inhibitors + Checkpoint Blockade	Exploratory	NCT04354246	Ongoing - Early Phase

OS, Overall Survival (in months); HR, Hazard Ratio; ORR, Objective Response Rate; DCR, Disease Control Rate; m, months.

In addition to targeting tumor cells directly, new strategies aim to modulate the TME to enhance anti-tumor immunity. This includes targeting immunosuppressive cells (e.g., regulatory T cells, myeloid-derived suppressor cells) and cytokines (e.g., TGF-β, IL-10) that inhibit the immune response (105).

Personalized medicine involves tailoring treatments to the specific genetic, molecular, and cellular characteristics of an individual’s cancer. This approach has the potential to improve the effectiveness of antibody therapies for HCC by ensuring that patients receive treatments most likely to benefit them. Identifying biomarkers that predict response to antibody therapies is critical for selecting the right patients for each treatment. For example, PD-L1 expression, TMB, and specific gene signatures can help identify patients who are likely to respond to ICIs (106). Comprehensive genomic profiling of tumors can reveal actionable mutations and alterations that can be targeted by specific antibody therapies. This approach allows for the customization of treatment plans based on the unique molecular characteristics of each patient’s cancer (107).

Adaptive trial designs, such as basket and umbrella trials, allow for the simultaneous evaluation of multiple treatments in different patient subgroups based on their molecular profiles. These innovative trial designs can accelerate the identification of effective therapies and improve patient outcomes (108).

Recent phase III clinical trials for unresectable HCC have continued to use sorafenib as the primary comparator, despite the establishment of atezolizumab plus bevacizumab as the SOC in the IMbrave150 trial. This approach is evident in trials such as HIMALAYA, which evaluated tremelimumab plus durvalumab versus sorafenib (109), and COSMIC-312, which assessed cabozantinib plus atezolizumab versus sorafenib (110). While these trials were designed before the results of IMbrave150 were available, their continued use of sorafenib as the control arm at the time of readout limits their generalizability and clinical impact.

The IMbrave150 trial demonstrated that atezolizumab plus bevacizumab significantly outperformed sorafenib in OS and PFS, with improved tolerability. Despite this, trials like HIMALAYA and COSMIC-312 continued to use sorafenib as the control arm, making their findings less applicable to current clinical practice. The HIMALAYA trial showed non-inferiority of the STRIDE regimen (tremelimumab plus durvalumab) versus sorafenib but did not evaluate its efficacy against atezolizumab plus bevacizumab. The COSMIC-312 trial failed to demonstrate OS superiority of cabozantinib plus atezolizumab versus sorafenib, raising doubts about its potential clinical role when the actual benchmark should have been atezolizumab plus bevacizumab. Without head-to-head comparisons to the true gold standard, clinicians are left uncertain about whether these therapies offer a real improvement or simply outperform an outdated regimen. Using an outdated comparator delays innovation because it does not challenge novel agents against the best available treatments. Trials with suboptimal control arms can misallocate resources and delay approval for more effective therapies that should be tested in a more competitive landscape. The persistent use of sorafenib as a comparator in recent HCC trials undermines clinical relevance, delays innovation, and hinders progress. Moving forward, trial designs must evolve to reflect the most current SOC, ensuring that new therapies are tested in the most competitive, clinically meaningful settings.

Combining antibody therapies with other treatment modalities can enhance their efficacy and overcome resistance mechanisms. Synergistic combinations can target different aspects of the tumor and its microenvironment, leading to improved therapeutic outcomes. Combining antibody therapies with locoregional treatments like TACE and RFA can enhance the overall anti-tumor effect. Locoregional therapies can reduce tumor burden, making the residual disease more susceptible to systemic treatments (111).

Radiotherapy has historically played a limited role in HCC treatment due to concerns about radiation-induced liver disease (RILD). However, advancements in stereotactic body radiotherapy (SBRT) have significantly improved precision, enabling its use in select patient populations, particularly those with portal vein tumor thrombosis (PVTT) (112). SBRT delivers high-dose radiation to tumor sites while minimizing liver toxicity, achieving local control rates of 70–90% in PVTT cases.

Radiotherapy is increasingly being explored in combination with ICIs and monoclonal antibodies, leveraging its ability to modulate the TME. Radiation induces tumor antigen release, promoting dendritic cell activation and antigen presentation. It upregulates PD-L1 expression, which may enhance response rates to anti-PD-1/PD-L1 checkpoint inhibitors (e.g., nivolumab, pembrolizumab, atezolizumab). The abscopal effect, where localized radiotherapy induces systemic anti-tumor immunity, has been observed in patients receiving ICIs (113, 114).

Several trials (e.g., RTOG-1112, NCT03316872) are evaluating SBRT in combination with PD-1/PD-L1 inhibitors to improve survival outcomes in advanced HCC (115). Early-phase results suggest increased response rates and prolonged progression-free survival compared to ICIs alone. Radiation therapy also induces hypoxia-driven VEGF upregulation, promoting angiogenesis and tumor progression. Combining SBRT with VEGF-targeting antibodies (e.g., bevacizumab, ramucirumab) may counteract this effect, improving local tumor control and reducing recurrence. IMbrave150 findings support the rationale for atezolizumab + bevacizumab + SBRT, which is currently under investigation (116). Combined antibody therapy and SBRT is challenged by the optimal dose and fractionation selection, the optimal treatment sequencing of antibody therapy relative to radiotherapy, and the selection of proper biomarkers to guide the treatment and select eligible patients.

While the potential for triple antibody therapy in HCC exists, current research is primarily focused on dual antibody combinations and integrating antibodies with other treatment modalities. Further studies are necessary to explore the safety, efficacy, and feasibility of triple antibody regimens in HCC treatment. A recent study has investigated the combination of transarterial chemoembolization (TACE), lenvatinib (a tyrosine kinase inhibitor), and anti-PD-1 antibodies, which has shown promising results in converting unresectable HCC to resectable status (117). Combining multiple antibodies increases the risk of immune-related adverse events, which necessitates a careful assessment of safety profiles through clinical trials to determine their effectiveness over existing therapies.

Nanotechnology offers innovative solutions for the targeted delivery of antibody therapies. Nanoparticles can be engineered to carry antibodies and release them in a controlled manner at the tumor site, enhancing the precision and effectiveness of the treatment (118).

## 8 Conclusion

Antibody-based therapies have revolutionized the treatment paradigm for hepatocellular carcinoma, offering new hope for patients with advanced disease. While significant progress has been made, continued research and innovation are essential to overcome current challenges and fully realize the potential of these therapies. By integrating cutting-edge technologies and personalized medicine approaches, the future holds promise for more effective, targeted, and accessible treatments for HCC, ultimately improving patient outcomes and survival rates.
